# Effect of intercropping medicinal herbs in *Pinus elliottii* plantation on soil properties and microbial community characteristics

**DOI:** 10.3389/fmicb.2026.1880144

**Published:** 2026-08-12

**Authors:** Shutong Sang, Wei Huang, Wendi Lan, Kaitai Yang, Baoling Chen, Suzhen Liu, Jun Liu, Dongxue Zhang, Fengqing Li, Xingliang Chen

**Affiliations:** 1 Experimental Center of Subtropical Forestry, Chinese Academy of Forestry, Fenyi, China; 2 Guangxi Forestry Research Institute, Nanning, China; 3 Guangxi Qipo State Forest Farm, Nanning, China

**Keywords:** enzyme activity, forest-medicinal herb intercropping pattern, microbial community, *Pinus elliottii*, soil nutrients

## Abstract

*Pinus elliottii* is an important afforestation species in the hilly regions of southern China, but long-term monoculture may lead to soil nutrient depletion and microbial community imbalance.

**Objective:**

This study aimed to evaluate the effects of different understory medicinal plant intercropping patterns on soil properties, enzyme activities, and soil microbial communities in *P. elliottii* plantations, and to identify suitable intercropping patterns for forest-medicinal plant compound management.

**Method:**

Three intercropping patterns were tested: *P. elliottii*-*Ficus simplicissima*-*Spatholobus suberectus* (PHS), *P. elliottii*-*Alpinia oxyphylla*-*S. suberectus* (PAS), *P. elliottii*-*Alpinia hainanensis*-*S. suberectus* (PSA), with a pure *P. elliottii* forest serving as control (CK). Soil nutrient dynamics and enzyme activities were determined, and soil microbial communities were analyzed using high-throughput sequencing.

**Result:**

Under the same intercropping pattern, soil nutrients in the 0–20 cm layer were higher than those in the 20–40 cm layer. In the 0–20 cm layer, all three intercropping patterns significantly reduced amylase activity compared to CK (*P* < 0.05). In the PAS pattern, acid phosphatase, neutral protease, and urease activities were significantly enhanced, with urease activity significantly higher in 0–20 cm than in the 20–40 cm layer. High-throughput sequencing revealed interactions among intercropping patterns, soil properties, and microbial communities. Intercropping significantly affected the structure, composition, and diversity of both soil bacterial and fungal communities. PSA showed pronounced effects on soil nutrient status and exhibited the highest bacterial and fungal ASV richness. Redundancy analysis (RDA) identified organic carbon (SOC) and total nitrogen (TN) as key drivers of microbial variation. Random forest and LEfSe analyses further identified key taxa, such as *Acidibacillus* and *Thermosporothrix*, as significantly associated with the intercropping effects. Functional prediction suggested that microorganisms may improve soil quality by regulating gene responses in “Metabolism of terpenoids and polyketides” and “Urea Cycle” pathways, as well as by secreting enzymes such as ferredoxin hydrogenase and β-mannosidase.

**Conclusion:**

Forest-medicinal plant intercropping improved soil quality and reshaped microbial communities in *P. elliottii* plantations. The PSA pattern showed the greatest potential for enhancing soil microecological functions and may serve as a suitable intercropping model for sustainable plantation management.

## 1 Introduction

Under-forest economy is a novel form of economic development that relies on forests, forestland, and their associated ecosystems. By making full use of the spatial advantages of forestland and scientifically planning three-dimensional land use under constrained resource conditions, this model integrates forestry production with under-forest industries, thereby promoting the coordinated development of ecosystem management, biodiversity conservation, and efficient resource utilization (Chen, 2022). Globally, understory agroforestry has emerged as a sustainable forest management strategy that reconciles ecological conservation and economic benefits. Forest-medicine intercropping has received increasing attention, particularly in China, where the under-forest economy has developed rapidly. According to statistics, by 2025, the total area of forestland utilized for under-forest economic activities in China had reached 43.33 million hectares, with the total output value exceeding 1 trillion yuan (National Forestry Grassland Administration, 2026). These developments highlight the growing importance of under-forest economic systems for balancing ecological conservation with sustainable forest-based production in China.

Agroforestry-based medicinal plant intercropping is one of the representative models of the under-forest economy. However, the effects of such systems on soil properties and microbial communities remain inconsistent and controversial across studies. Some researchers have reported negative or deleterious effects, including intensified interspecific competition, soil degradation, reduced enzyme activities, and declined microbial diversity (Wang et al., 2020; Li et al., 2001). In contrast, several other investigations have found no significant influence of forest-medicine intercropping on soil physicochemical characteristics, microbial alpha diversity, or community stability (Liu et al., 2024a,b; Zhou et al., 2022). These conflicting results reveal the context-dependent complexity of belowground responses and suggest that the soil microecological effects of forest-medicine intercropping may depend on medicinal plant species and belowground interactions. However, whether different intercropping patterns induce consistent or species-specific responses remains unclear, hindering the scientific selection of suitable intercropping patterns.

Nevertheless, rationally designed forest-medicine intercropping can optimize resource niche partitioning, ameliorate stand microenvironments, and improve soil fertility and microbial conditions (Wu et al., 2023; Xue et al., 2023; Curtright Andrew and Tiemann Lisa, 2021). Such agroforestry practices have been associated with increased microbial diversity and more complex community structure and may modulate soil biogeochemical cycles to mitigate nutrient loss (Lian et al., 2019; Zhang et al., 2021; Wang et al., 2025). Soil microorganisms are integral to below-ground ecological processes and plant performance across plantation ecosystems. However, microbial feedbacks are highly species-dependent, as documented in multiple agroforestry scenarios involving various tree and herbaceous species (Li et al., 2023; Wang et al., 2024; Lu et al., 2024). Relevant studies from North America have further shown that diversified understory cultivation could reshape soil microbial profiles and enzyme characteristics in forest plantations (Calderón et al., 2016). However, evidence specific to *Pinus elliottii* plantations remains limited, and few studies have simultaneously examined soil properties, enzyme activities, and bacterial and fungal communities to elucidate their coordinated responses to different medicinal plant intercropping patterns. Moreover, whether these responses vary with soil depth remains poorly understood.

Given the unresolved controversies surrounding the effects of forest-medicine intercropping on soil microecology and the limited evidence specific to *Pinus elliottii* plantations, this study addresses two core research questions. First, we examined whether intercropping different medicinal plants under *Pinus elliottii* altered soil nutrient contents and enzyme activities, thereby forming a beneficial soil microecological feedback. Second, we evaluated whether different forest-medicine intercropping patterns reshaped microbial communities characteristics and stability, and whether these effects varied among medicinal plant species. Accordingly, we hypothesized that different medicinal plant intercropping patterns would differentially alter soil nutrient status, enzyme activities, and microbial community characteristics, and that these responses would vary with soil depth.

To address the above research questions, we investigated typical understory medicinal plant intercropping patterns in Pinus elliottii plantations, using pure *P. elliottii* plantations as the control. Three core research objectives were established: (1) to explore differences in the activities of key soil enzymes among various forest-medicine intercropping patterns and clarify the linkages between microbial metabolic pathways and soil enzyme activities; (2) to characterize the responses and co-occurrence relationships of bacterial and fungal communities under different intercropping patterns; and (3) to clarify the relationships among microbial metabolic pathways, soil enzyme activities, and soil physicochemical properties, elucidate the potential mechanisms by which forest-medicine intercropping regulates soil microecology in *P. elliottii* plantations, and provide theoretical support for selecting suitable intercropping patterns and promoting the coordinated development of the understory economy and ecological conservation in *P. elliottii* forests.

## 2 Materials and methods

### 2.1 Materials

#### 2.1.1 Study area and sample collection

The experimental forest site was located at Qipo State-owned Forest Farm, Guangxi Zhuang Autonomous Region, China (22 °41′-22 °52′N, 108 °02′-108 °09′E). The area had a humid subtropical monsoon climate, characterized by abundant sunlight and plentiful rainfall, with a mean annual temperature of 21.6 °C and mean annual precipitation of 1,300 mm. *P. elliottii* stand in the study area was 49 years old, with a density of 600 plants·hm^−2^, a tree height ranging from 23 to 41 m, and a diameter at breast height ranging from 28 to 46 cm. The understory soil was lateritic red soil and strongly acidic (pH 3.25–4.47).

Samples were collected from three different intercropping patterns and one control treatment. The three intercropping treatments followed a multi-layer configuration, with trees in the upper layer, vine medicinal plants in the middle layer, and herbaceous medicinal plants in the lower layer. The PHS treatment consisted of *P. elliottii, Ficus simplicissima* Lour., and *Spatholobus suberectus* Dunn; the PAS treatment consisted of *P. elliottii, Alpinia oxyphylla* Miq. and *S. suberectus*; and the PSA treatment consisted of *P. elliottii, Alpinia hainanensis* and *S. suberectus*. The control treatment (CK) was a pure *P. elliottii* plantation.

Soil samples were collected in May 2025. Using the five-point sampling method, three sampling points were randomly selected in each plot, and soil was collected by the soil profile method at depths of 0–20 cm and 20–40 cm. Plant roots and stones were removed from the soil samples, which were then passed through a 2 mm sieve and thoroughly homogenized. Soil collected from two adjacent sampling points was combined to form one composite sample, and three replicate samples were obtained for each soil layer. Each soil sample was then divided into two portions: one portion was air-dried indoors for analysis of soil chemical properties, while the other was preserved on dry ice and transported to the laboratory for determination of soil enzyme activities and microbial characteristics, with the measurements conducted by Majorbio Bio-Pharm Technology Co., Ltd. (Shanghai, China; Li et al., 2026).

### 2.2 Methods

#### 2.2.1 Determination of soil chemical properties

The soil organic carbon content was determined using the potassium dichromate oxidation–external heating method (LY/T1237–1999, National Forestry Standard of China). Soil total nitrogen was determined by the digestion–indophenol blue colorimetric method with a mixed sulfuric acid reagent (LY/T1228–2015, National Forestry Standard of China). Soil total phosphorus was measured using the sodium hydroxide fusion–molybdenum antimony anti-colorimetric method (LY/T1232–2015, National Forestry Standard of China).

#### 2.2.2 Determination of soil enzyme activities

The activities of soil acid phosphatase (S-ACP), amylase (AL), urease (UE), nitrate reductase (NR), sucrase (SC), and neutral protease (NP) were determined using commercial assay kits purchased from Solarbio Science & Technology Co., Ltd. (Beijing, China). All enzyme activities were quantified spectrophotometrically. The activity unit of S-ACP was defined as 1 nmol phenol released daily per gram of soil at 37 °C. For AL, a single activity unit corresponded to 1 μmol reducing sugar synthesized per gram of soil within 1 day. UE activity was calculated according to the daily production of 1 μg NH_3_-N per gram of soil. The formation of 1 μmol NO_2_? per gram of soil per day represented one activity unit of NR. The unit of SC activity referred to 1 mg reducing sugar accumulated in per gram of soil per day under 37 °C. In terms of NP, one activity unit was equivalent to 1 μmol tyrosine generated daily per gram of soil.

#### 2.2.3 Determination of soil microbial diversity

Soil microbial community DNA was extracted using the E.Z.N.A.^®^ Soil DNA Kit (Omega Bio-tek, Norcross, GA, USA). DNA extraction quality was assessed by 1% agarose gel electrophoresis, and DNA concentration and purity were determined using a NanoDrop 2000 spectrophotometer (Thermo Scientific, USA). The extracted DNA was used as the template for PCR amplification. For fungi, the V5–V7 variable region was amplified using the primers ITS3F (5′-GCATCGATGAAGAACGCAGC-3′) and ITS4R (5′-TCCTCCGCTTATTGATATGC-3′). For bacteria, the V3–V4 variable region of the 16S rRNA gene was amplified using the primers 338F (5′-ACTCCTACGGGAGGCAGCAG-3′) and 806R (5′-GGACTACHVGGGTWTCTAAT-3′). The PCR reaction mixture (20 μL) consisted of 10 μL of 2 × Pro Taq, 0.8 μL of each forward and reverse primer, 10 ng of DNA template, and ddH_2_O added to a final volume of 20 μL. The PCR program was as follows: initial denaturation at 95 °C for 3 min; 35 cycles of denaturation at 95 °C for 30 s, annealing at 55 °C for 30 s, and extension at 72 °C for 45 s; followed by a final extension at 72 °C for 10 min, and storage at 4 °C. PCR amplification was performed using an ABI GeneAmp^®^ 9700 thermal cycler. PCR products were recovered using 2% agarose gel electrophoresis, purified with a PCR Clean-Up Kit (YuHua, China), and quantified using a Qubit 4.0 fluorometer (Thermo Fisher Scientific, USA).

Purified PCR products were used for library construction with the NEXTFLEX Rapid DNA-Seq Kit as follows: (1) adapter ligation; (2) removal of self-ligated adapter fragments using magnetic bead selection; (3) enrichment of library templates by PCR amplification; and (4) recovery of PCR products using magnetic beads to obtain the final library. Sequencing was then carried out on the Illumina NextSeq 2000 platform by Majorbio Bio-Pharm Technology Co., Ltd. (Shanghai, China). Raw sequencing reads were quality-filtered using fastp (Chen et al., 2018; version 0.19.6), and paired-end reads were merged using FLASH (Magoč and Salzberg, 2011; version 1.2.11) to obtain valid sequences, which were further used to generate amplicon sequence variants (ASVs) representing the true sequence information in each sample.

### 2.3 Data processing and statistical analysis

Data on soil physicochemical properties were collected and organized using Excel 2021. One-way analysis of variance (ANOVA) and significance tests at the *P* < 0.05 level were performed using SPSS 27.0 to evaluate differences in soil physicochemical properties and soil enzyme activities among different treatment patterns. The effects of intercropping pattern, soil depth, and their interaction on soil property indicators were assessed using two-way analysis of variance (two-way ANOVA). Statistical significance was determined at *P* < 0.05. Pearson correlation analysis was used to examine the relationships among soil physicochemical properties, soil enzyme activities, and microbial diversity under different patterns. All data were presented as the mean ± standard deviation. High-throughput sequencing data were analyzed on the Majorbio Cloud Platform (https://cloud.majorbio.com). Alpha diversity indices, including Chao, Sobs, Shannon, and Coverage, were used to evaluate microbial community diversity, and intergroup differences were assessed using the Wilcoxon rank-sum test (Li et al., 2026). Non-metric Multidimensional Scaling (NMDS) was applied for visualizing the overall dissimilarity of microbial communities across treatments, and analysis of similarities (ANOSIM) was conducted to test the statistical significance of intergroup community differences based on Bray–Curtis distance matrices. Differences in bacterial and fungal community composition were described based on relative abundance at the phylum level. Spearman correlation analysis was conducted to explore the associations among soil physicochemical properties, soil enzyme activities, and microbial abundance, while Mantel tests were used to assess the direct or indirect effects of key factors on microbial communities (Mu et al., 2024). Distance-based redundancy analysis (db-RDA) based on Bray–Curtis distance was performed to investigate the effects of soil physicochemical properties and soil enzyme activities on the composition of soil bacterial and fungal communities. In the correlation analyses, statistical significance was defined at *P* < 0.05. Microbiological data were processed and visualized using Tutools (https://www.cloudtutu.com) and Wekemo Bioincloud (https://www.bioincloud.tech). The resulting node and edge files were then imported into Gephi for network visualization, in which nodes were colored according to microbial phylum-level classification and node size was proportional to the abundance of the corresponding taxon (Ren et al., 2022). Key microorganisms were visualized in Origin 2022 based on Zi–Pi values. Figures were further refined using Adobe Illustrator.

## 3 Results and analysis

### 3.1 Differences in soil nutrient contents under different medicinal plant intercropping patterns

**Figure 1:**
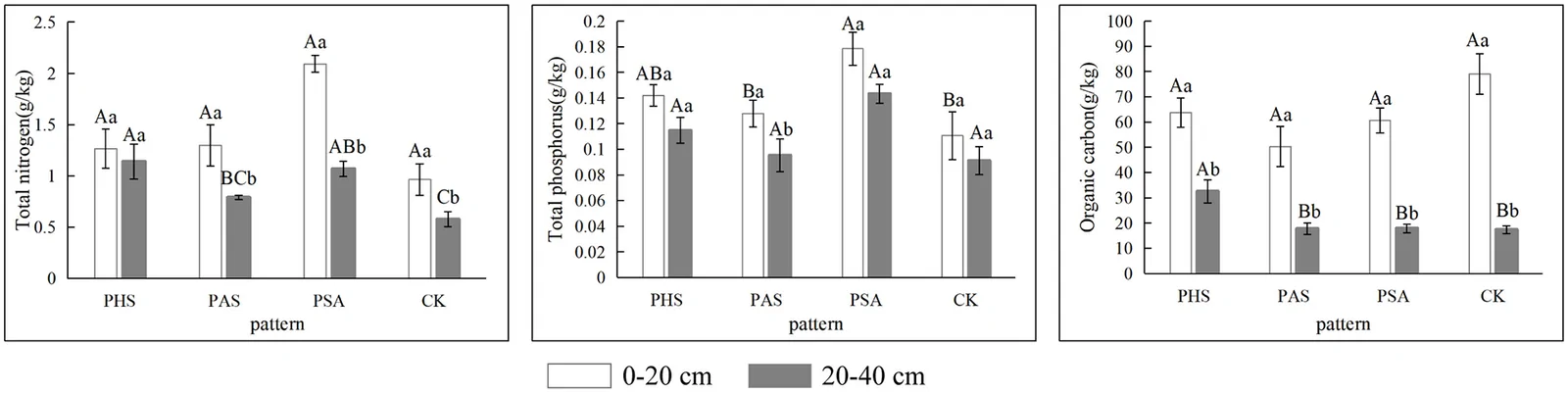
The nutrient contents of soils in different modes and soil horizons. Different lowercase letters indicate significant differences among different soil layers in the same stand pattern; different uppercase letters indicate significant differences among different patterns in the same soil layer, *P* < 0.05. PHS, *Pinus elliottii-Ficus simplicissima-Spatholobus suberectus*; PAS, *Pinus elliottii-Alpinia oxyphylla-Spatholobus suberectus*; PSA, *Pinus elliottii-Alpinia hainanensis-Spatholobus suberectus*; CK, pure *Pinus elliottii* plantation.

Intercropping *P. elliottii* with different medicinal plants had significant effects on soil nutrient contents under different intercropping patterns and soil depths (Figure 1). In the 0–20 cm soil layer, no significant differences were observed in soil total nitrogen (TN) or organic carbon (SOC) contents between the intercropping treatments and the control. However, the soil total phosphorus (TP) content in the PSA treatment was significantly higher than that in the control (*P* < 0.05). In the 20–40 cm soil layer, TN content differed significantly among intercropping patterns, with the PHS and PSA treatments showing significantly higher values than the control (*P* < 0.05). No significant differences were found in TP content between the three intercropping treatments and the pure *P. elliottii* plantation, although the overall trend was PSA > PHS > PAS > CK. The SOC content in the PHS treatment was significantly higher than that in the pure *P. elliottii* plantation (*P* < 0.05). Comparisons between soil layers within the same treatment showed that the contents of SOC, TN, and TP in all treatments decreased with increasing soil depth. Specifically, SOC in the PHS treatment; SOC, TN, and TP in the PAS treatment; SOC and TN in the PSA treatment; and SOC and TN in the pure *P. elliottii* plantation all showed significantly higher values in the upper soil layer than in the lower soil layer (*P* < 0.05).

### 3.2 Differences in soil enzyme activities under different medicinal plant intercropping patterns

**Figure 2:**
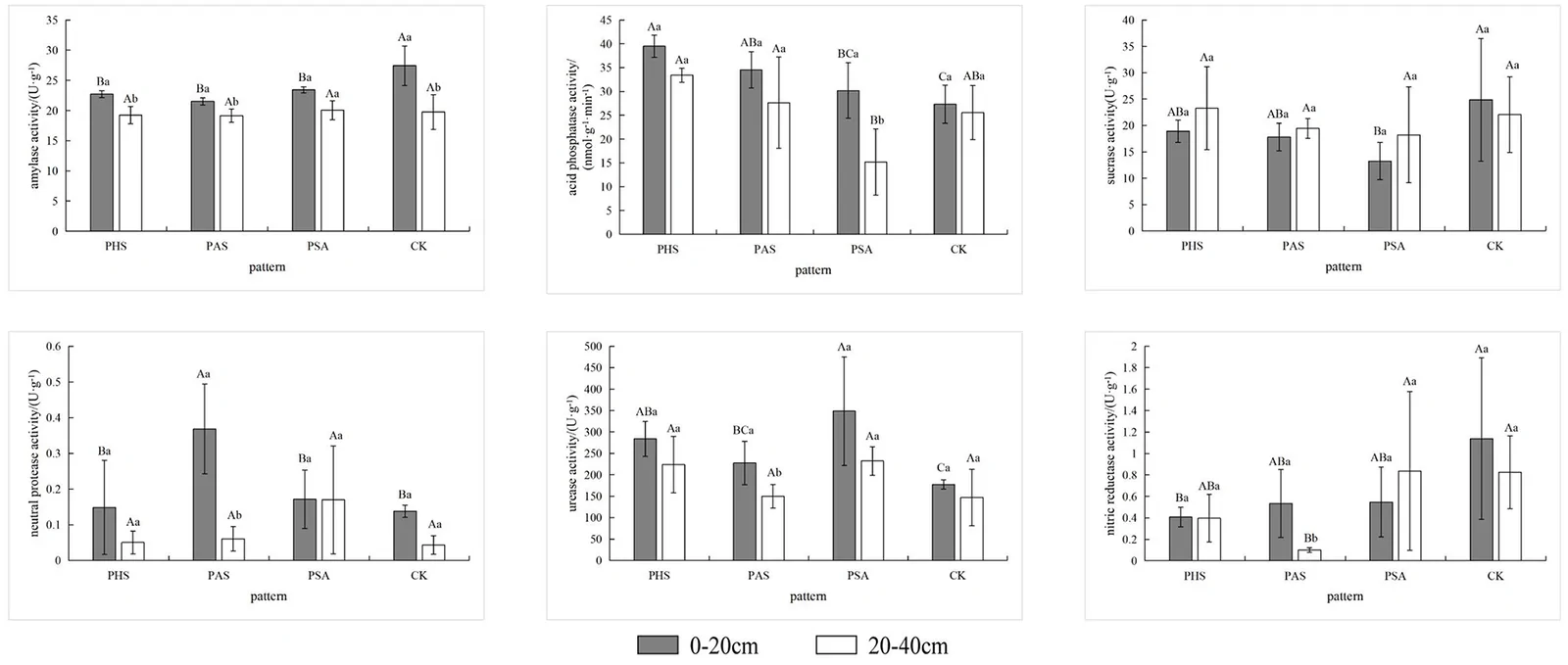
Enzyme activities of soils in different patterns and soil depth. Different lowercase letters indicate significant differences among different soil layers in the same stand pattern; different uppercase letters indicate significant differences among different patterns in the same soil layer, *P* < 0.05. PHS, *Pinus elliottii-Ficus simplicissima-Spatholobus suberectus*; PAS, *Pinus elliottii-Alpinia oxyphylla-Spatholobus suberectus*; PSA, *Pinus elliottii-Alpinia hainanensis-Spatholobus suberectus*; CK, pure *Pinus elliottii* plantation.

Different intercropping patterns and soil depths affected the activities of the six soil enzymes to varying degrees (Figure 2). In the 0–20 cm soil layer, amylase activity was significantly lower in all three intercropping treatments than in the pure *P. elliottii* plantation (CK; *P* < 0.05). Acid phosphatase activity was significantly higher in the PHS and PAS treatments than in CK (*P* < 0.05). Sucrase activity was lower in all intercropping treatments than in CK, whereas neutral protease activity in the PAS treatment (0.37 ± 0.13 U/g) was significantly higher than that in the other three treatments (*P* < 0.05). Urease activity in the PHS and PSA treatments was significantly higher than that in CK (*P* < 0.05). Nitrate reductase activity in the PHS treatment (0.41 ± 0.09 U/g) was significantly lower than that in CK (*P* < 0.05), while no significant differences were found between the other treatments and CK. In the 20–40 cm soil layer, the patterns of variation in enzyme activities changed to some extent. No significant differences were observed among treatments in amylase, urease, or neutral protease activity. Acid phosphatase activity in the PSA treatment (15.16 ± 6.96 nmol/g/min) was significantly lower than that in CK (*P* < 0.05). Sucrase activity followed the order PHS > CK > PAS > PSA. In terms of vertical distribution, except for amylase activity in the PSA treatment, the PHS, PAS, and CK treatments all showed significantly higher amylase activity in the upper soil layer than in the lower soil layer (*P* < 0.05). Acid phosphatase activity was significantly higher in the upper soil layer than in the lower soil layer only in the PSA treatment (*P* < 0.05). Neutral protease, urease, and nitrate reductase activities were significantly higher in the upper soil layer than in the lower soil layer only in the PAS treatment (*P* < 0.05).

### 3.3 Effects of different intercropping patterns and soil depths on soil properties

The two-way ANOVA results showed that the effects of intercropping pattern and soil depth on soil properties varied among indicators (Table 1). Soil depth was the primary factor driving the differentiation of soil properties, with highly significant effects on N, C, AL, S-ACP, NP, and UE (*P* < 0.01), indicating pronounced differences in soil nutrient contents and nutrient transformation-related enzyme activities between soil layers and a clear vertical distribution pattern. By contrast, intercropping pattern significantly affected only soil enzyme activity indicators, including S-ACP, UE, and NR (*P* < 0.05), suggesting that intercropping systems may regulate soil nutrient transformation mainly by altering soil enzyme activities. For interaction effects, significant intercropping pattern × soil depth interactions were detected only for AL and NP (*P* < 0.05), implying that the responses of these two indicators to intercropping pattern depended on soil depth. No significant interaction effects were observed for the other soil indicators, indicating that most soil physicochemical and enzymatic properties were primarily influenced by the main effects of intercropping pattern or soil depth rather than by their combined effects.

**Table 1:** Two-way ANOVA examining the effects of intercropping patterns and soil depths on soil physicochemical properties.

Soil indicators	Intercropping patterns		Different soil depths		Pattern × Soil depth	
	F	*P*	F	*P*	F	*P*
TN	2.736	0.094	40.471	<0.001	1.039	0.414
TP	1.987	0.174	0.129	0.726	0.801	0.519
SOC	0.886	0.478	67.096	<0.001	1.413	0.291
AL	3.079	0.072	139.713	<0.001	9.526	0.002
S-ACP	7.195	0.006	17.376	0.002	2.365	0.127
SC	1.555	0.256	0.999	0.339	0.684	0.580
NP	2.471	0.116	15.726	0.002	4.507	0.027
UE	4.902	0.021	12.742	0.004	0.779	0.530
NR	9.304	0.002	0.366	0.557	0.728	0.557

### 3.4 Composition and diversity of soil microbial communities under different medicinal plant intercropping patterns

**Figure 3:**
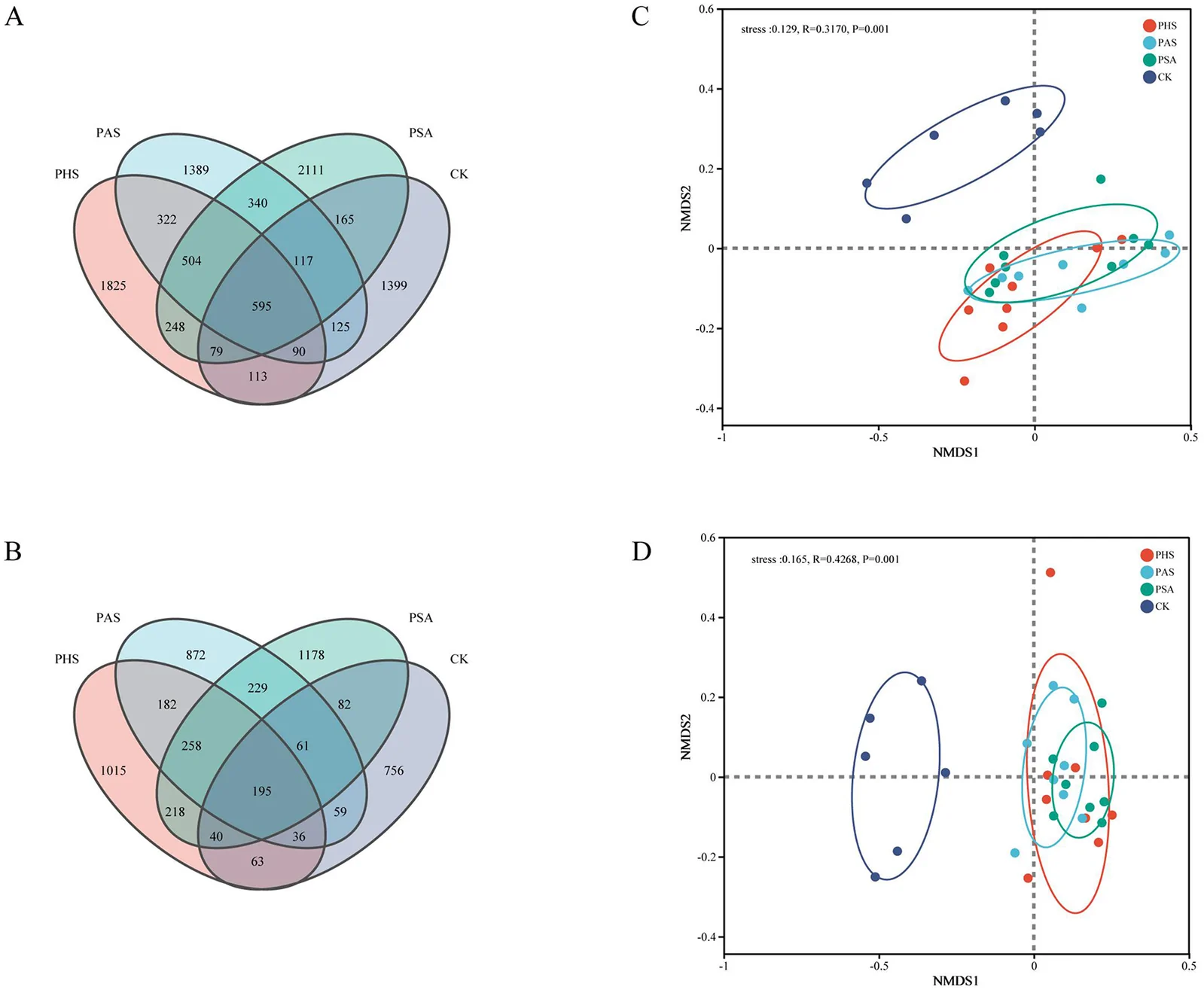
Microbial community composition and β-diversity: **(A)** Venn diagram of bacterial community; **(B)** Venn diagram of fungal community; **(C)** non-metric multidimensional scaling of bacterial community; **(D)** non-metric multidimensional scaling of fungal community.

At the ASV level, the bacterial communities shared 595 ASVs across the four treatments (Figures 3A, B). The numbers of unique bacterial ASVs in the PHS, PAS, PSA, and CK treatments were 1,825, 1,389, 2,111, and 1,399, respectively. For fungal communities, 195 ASVs were shared among the four treatments, whereas the numbers of unique fungal ASVs were 1,015, 872, 1,178, and 756 in the PHS, PAS, PSA, and CK treatments, respectively. Overall, bacterial communities exhibited higher ASV richness than fungal communities. The PSA treatment showed the highest ASV richness for both bacteria and fungi, whereas the CK treatment had the lowest richness.

**Figure 4:**
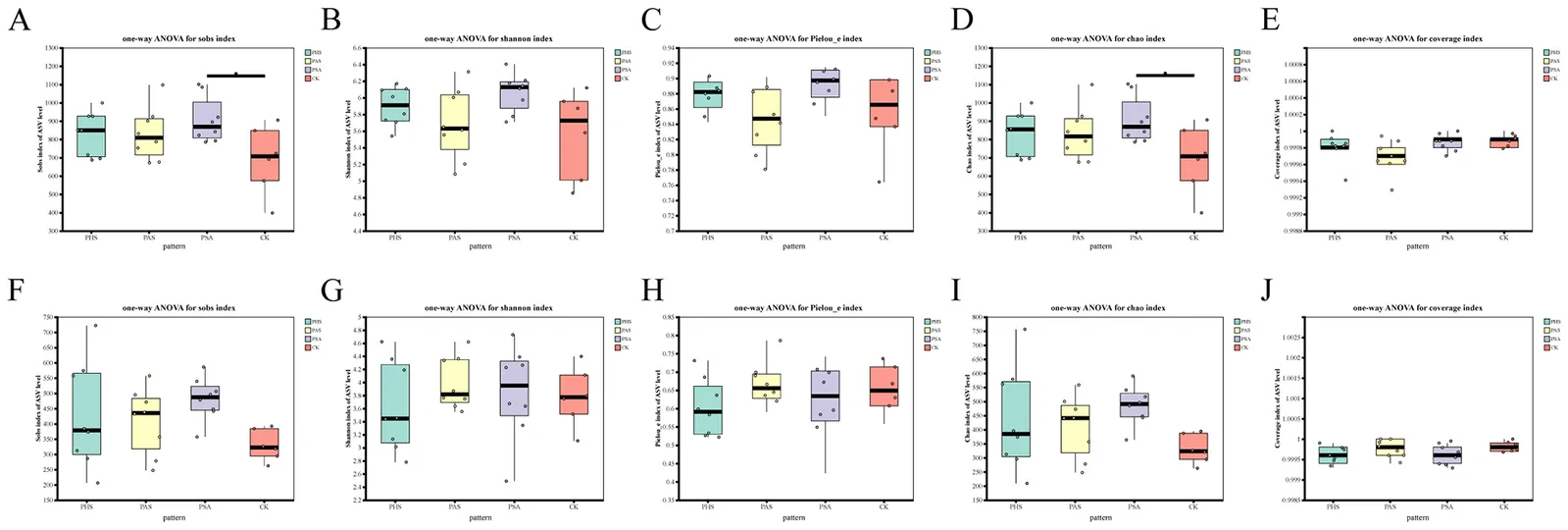
Alpha diversity of microbial communities: **(A–E)** alpha diversity of bacteria; **(F–J)** alpha diversity of fungi.

Alpha diversity of bacterial and fungal communities was evaluated using indices such as Chao (Figure 4). Among the bacterial communities, all diversity indices were highest in the PSA treatment. Significant differences were observed between the PSA and CK treatments in the bacterial Chao richness index and the Sobs observed richness index (*P* < 0.05), whereas no significant differences were detected for the other bacterial indices among treatments. For fungal communities, species richness was highest in the PSA treatment, while community diversity and evenness were highest in the PAS treatment. However, none of the fungal alpha diversity indices differed significantly among the four treatments. Coverage values were all 1.00, indicating that both bacterial and fungal taxa in the samples were adequately detected and that the sequencing results reliably reflected the biological characteristics of the microbial communities.

NMDS ordination visually exhibited distinct grouping patterns of microbial communities across treatments (Figures 3C, D). The stress values were 0.129 and 0.165, both below 0.2, which demonstrated that the ordination model had good fitting performance and reliable results. Samples from each treatment were enclosed within separate 95% confidence ellipses; the ellipses of the three intercropping regimes did not overlap with the ellipse of CK, presenting obvious visual separation between intercropping groups and the control. Subsequent ANOSIM analysis further confirmed that the community compositional dissimilarity among treatments reached a statistically significant level (*P* = 0.001). In contrast, the confidence ellipses of PHS, PAS and PSA largely overlapped for both bacterial and fungal datasets, implying relatively similar community structures among the three intercropping patterns.

**Figure 5:**
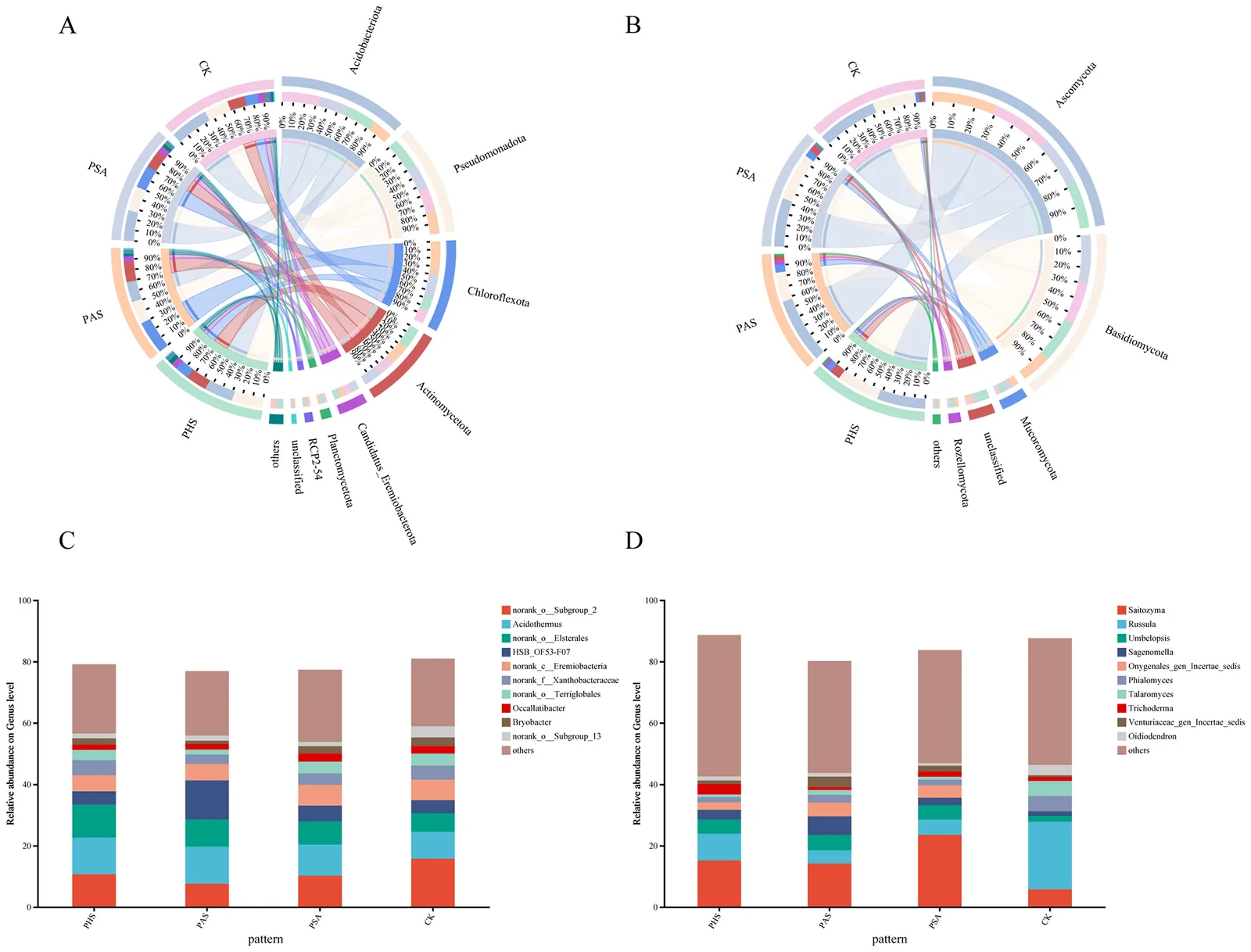
Microbial community composition: **(A)** Chord diagram of bacterial community at the phylum level; **(B)** Chord diagram of fungal community at the phylum level; **(C)** relative abundance of bacterial community at the genus level; **(D)** relative abundance of fungal community at the genus level. The relative abundance at the genus level showed the top 10 dominant genera.

The Circos plots were used to visualize the taxonomic composition of microbial communities at the phylum level (Figures 5A, B). The bacterial communities were mainly composed of Acidobacteriota, Pseudomonadota, Chloroflexota, Actinomycetota, Candidatus_Eremiobacterota, Planctomycetota, and RCP2-54. Compared with CK, the relative abundance of Chloroflexota increased by 1%, 24%, and 11% in the PHS, PAS, and PSA treatments, respectively. The dominant fungal phyla were generally similar among treatments and were mainly composed of Ascomycota, Basidiomycota, Mucoromycota, and Rozellomycota, accounting for more than 88% of the total fungal community. Nevertheless, the relative abundances of these fungal phyla varied among treatments.

At the genus level, percentage bar plots were used to compare the top 10 genera in terms of relative abundance (Figures 5C, D). Excluding unclassified taxa, the dominant bacterial genus in the PHS, PSA, and CK soils was *Acidothermus*, with relative abundances of 11.94%, 12.13%, and 8.78%, respectively, whereas the dominant genus in PAS was HSB_OF53-F07 (12.76%). Differences in fungal community composition were also observed among treatments at the genus level. Unclassified taxa accounted for a large proportion of the fungal community. The dominant fungal genus in the PHS, PAS, and PSA soils was *Saitozyma*, with relative abundances of 15.21%, 14.17%, and 23.54%, respectively, whereas *Russula* was dominant in CK (22.03%).

**Figure 6:**
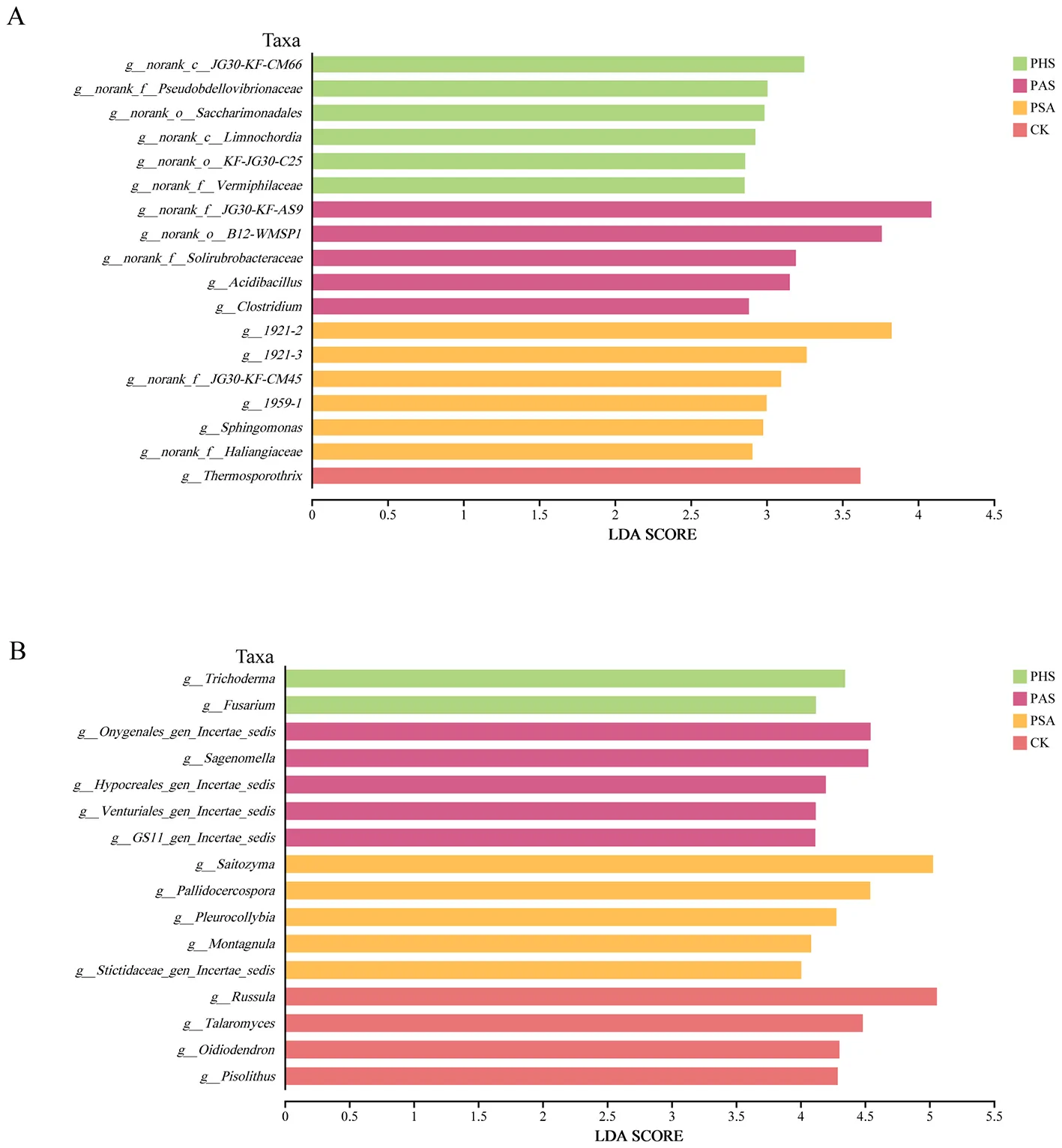
LEfSe analysis of microbial community composition: **(A)** LEfSe analysis of bacterial community composition (LDA > 2); **(B)** LEfSe analysis of fungal community composition (LDA > 4).

LEfSe analysis was further performed to identify differentially enriched bacterial (LDA > 2) and fungal taxa (LDA > 4) at the genus level (Figure 6). Excluding unclassified taxa, *Acidibacillus* and *Clostridium* were significantly enriched in the PAS treatment (*P* < 0.05), while 1921-2, 1921-3, *Sphingomonas*, and 1959-1 were significantly enriched in the PSA treatment (*P* < 0.05). *Thermosporothrix* was more strongly enriched in CK (*P* < 0.05). In the fungal community, *Trichoderma* and *Fusarium* were significantly enriched in the PHS treatment (*P* < 0.05); Onygenales_gen_Incertae_sedis, *Sagenomella*, Hypocreales_gen_Incertae_sedis, Venturiales_gen_Incertae_sedis, and GS11_gen_Incertae_sedis were significantly enriched in the PAS treatment (*P* < 0.05); *Saitozyma, Pallidocercospora, Pleurocollybia, Montagnula*, and Stictidaceae_gen_Incertae_sedis were significantly enriched in the PSA treatment (*P* < 0.05); and *Russula, Talaromyces, Oidiodendron*, and *Pisolithus* were significantly enriched in the CK treatment (*P* < 0.05).

### 3.5 Co-occurrence network analysis of soil microbial communities under different medicinal plant intercropping patterns

**Figure 7:**
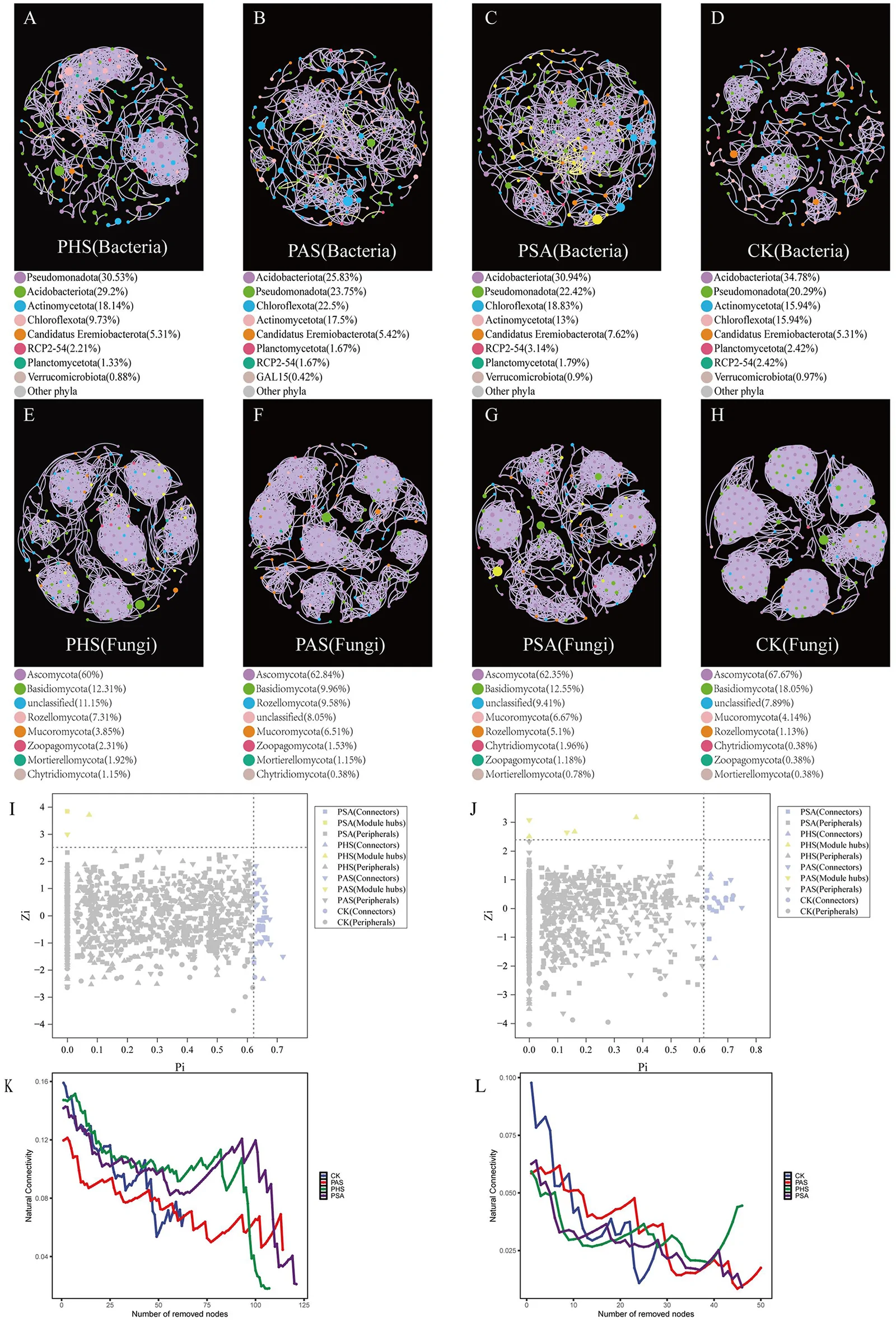
Microbial co-occurrence network analysis: **(A–D)** co-occurrence network diagrams for bacterial communities; **(E–H)** co-occurrence network diagrams for fungal communities; **(I)** Zi–Pi plot for bacterial network; **(J)** Zi–Pi plot for fungal network; **(K)** robustness analysis of bacterial networks; **(L)** robustness analysis of fungal networks.

Separate co-occurrence network analyses were performed for bacterial and fungal communities based on ASV-level microbial community data to clarify the interactions among ASVs in soils under different medicinal plant intercropping patterns (Figures 7A–H). The topological properties of the bacterial networks (Table 2) showed that the PHS treatment contained 226 nodes and 932 edges, of which 99.36% were positive correlations. The PAS treatment contained 240 nodes and 677 edges, with 96.16% of the edges showing positive correlations. The PSA treatment contained 223 nodes and 813 edges, among which 95.08% were positive correlations. The CK treatment contained 207 nodes and 568 edges, with a positive correlation ratio of 99.65%. The PHS treatment exhibited the highest average clustering coefficient (0.281), indicating the strongest degree of interconnection within the bacterial network. Robustness analysis further demonstrated that the bacterial network in the PHS treatment was the most stable and complex (Figure 7K), followed by the PSA treatment. The topological properties of the fungal networks (Table 2) showed that the PHS treatment contained 260 nodes and 1,879 edges, of which 99.95% were positive correlations. The PAS treatment contained 261 nodes and 1,626 edges, all of which were positive correlations. The PSA treatment contained 255 nodes and 1,486 edges, with 99.87% of the edges showing positive correlations. The CK treatment contained 266 nodes and 3,147 edges, all of which were positive correlations. These results indicate extensive ASV interactions within the fungal networks. Robustness analysis showed that the PAS treatment had the highest network stability (Figure 7L), whereas the CK treatment had the lowest stability.

**Table 2:** Topological parameters of co-occurrence networks of microbial communities.

Network index	Bacteria				Fungi			
	PHS	PAS	PSA	CK	PHS	PAS	PSA	CK
Edge	932	677	813	568	1,879	1,626	1,486	3,147
Node	226	240	223	207	260	261	255	266
Positive correlation	99.36%	96.16%	95.08%	99.65%	99.95%	100%	99.87%	100%
Negative correlation	0.64%	3.84%	4.92%	0.35%	0.05%	0	0.13%	0
Network density	0.018	0.012	0.016	0.013	0.028	0.024	0.023	0.045
Average clustering coefficient	0.281	0.241	0.25	0.276	0.358	0.353	0.342	0.425
Modularity	0.7	0.763	0.692	0.856	0.825	0.811	0.836	0.823
Average degree	4.124	2.821	3.646	2.744	7.227	6.23	5.827	11.831
Average path length	1.761	2.53	2.636	1.27	1.898	1.88	1.747	1.348
Number of communities	46	29	34	55	45	65	57	84
Number of weakly connected components	21	15	5	35	8	10	11	7
Number of strongly connected components	226	240	223	207	260	261	255	266

PHS, Pinus elliottii-Ficus simplicissima-Spatholobus suberectus; PAS, Pinus elliottii-Alpinia oxyphylla-Spatholobus suberectus; PSA, Pinus elliottii-Alpinia hainanensis-Spatholobus suberectus; CK, pure Pinus elliottii plantation.

Based on network topological characteristics, the microbial communities were further classified into four node categories, namely Module hubs, Network hubs, Connectors, and Peripherals (Figures 7I, J). The bacterial network contained 3 Module hubs and 40 Connectors, whereas the fungal network contained 5 Module hubs and 21 Connectors. The key bacterial genera included *Edaphobacter, Acidothermus*, and *Acidibacter*, while the key fungal genera included *Mortierella, Pisolithus*, and *Humicola*.

### 3.6 Correlation analysis

**Figure 8:**
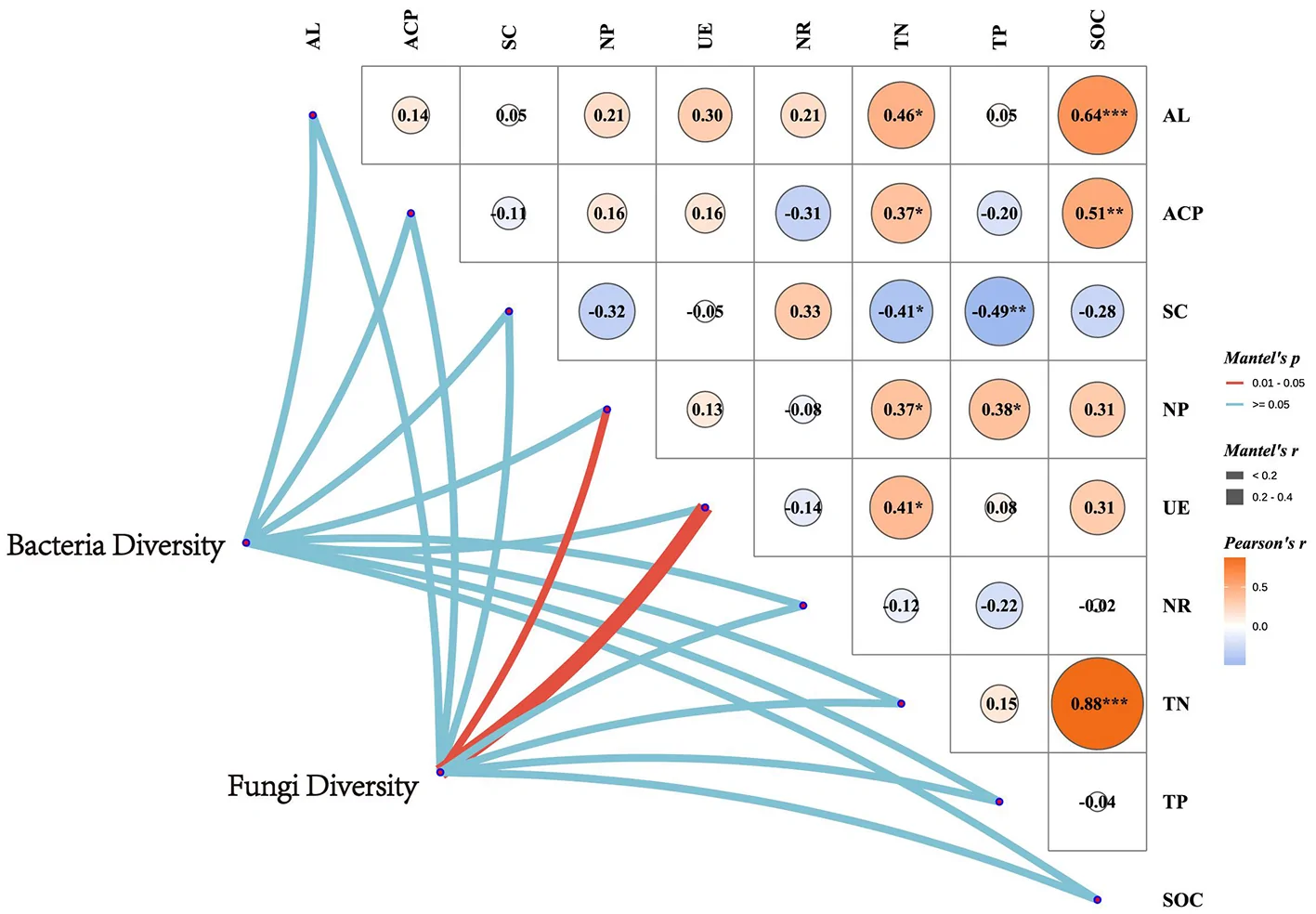
Correlation analysis between soil microbial diversity indexes and environmental factors. Red indicates positive correlations, blue indicated negative correlations. The width of connecting lines corresponds to distance-related statistical measures, while the color intensity reflects the magnitude of the positive/ negative correlations. Asterisks in color blocks denote statistical significance. *P < 0.05, **P < 0.01, ***P < 0.001.

Mantel tests were conducted to evaluate the relationships between microbial diversity and soil chemical properties (Figure 8). The correlation heatmap showed no significant associations between bacterial community diversity and soil parameters, suggesting that bacterial diversity was less affected by soil properties. In contrast, fungal community diversity was significantly correlated with neutral protease and urease activities (*P* < 0.05), indicating that fungal diversity may be more sensitive to variations in soil enzyme activity. In addition, soil SOC content was significantly and positively correlated with TN content and amylase activity (*P* < 0.05).

**Figure 9:**
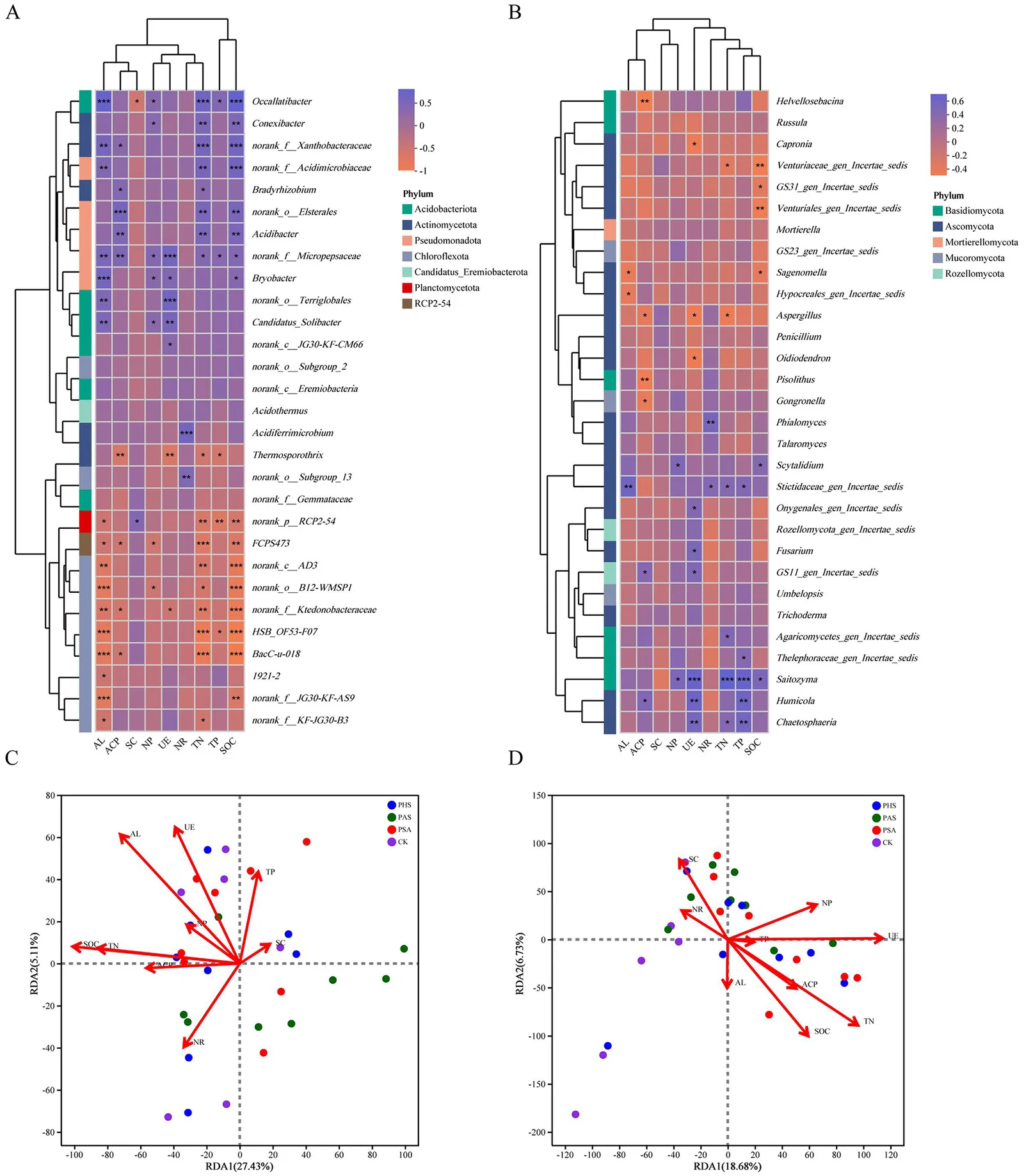
**(A)** Correlation analysis between bacterial genera and soil enzyme activities; **(B)** correlation analysis between fungal genera and soil enzyme activities; **(C)** Redundancy analysis of bacterial communities; **(D)** redundancy analysis of fungal communities. PAS, *Pinus elliottii-Alpinia oxyphylla-Spatholobus suberectus*; PSA, *Pinus elliottii-Alpinia hainanensis-Spatholobus suberectus*; CK, pure *Pinus elliottii* plantation.

The correlation heatmaps (Figures 9A, B) further showed significant associations between the relative abundances of the selected key bacterial and fungal genera and soil properties (*P* < 0.05). Among the bacterial genera, the relative abundances of *Occallatibacter*, HSB_OF53-F07, and BacC-u-018 were significantly and positively correlated with soil SOC, TN content, and amylase activity (*P* < 0.05). The relative abundance of *Acidiferrimicrobium* was significantly and positively correlated with soil nitrate reductase activity (*P* < 0.05). The relative abundance of *Bryobacter* was significantly and positively correlated with soil amylase activity (*P* < 0.05), whereas that of FCPS473 was significantly and positively correlated with soil TN content (*P* < 0.05). Among the fungal genera, the relative abundance of *Saitozyma* was significantly and positively correlated with soil TP, TN content, and urease activity (*P* < 0.05). Redundancy analysis (RDA) further confirmed that soil SOC and TN contents were the key factors shaping both bacterial and fungal community composition (Figures 9C, D). In addition, amylase and urease activities were important factors influencing bacterial community composition, whereas urease activity was a key factor affecting fungal community composition.

### 3.7 Functional prediction of enzymes and metabolic pathways

**Figure 10:**
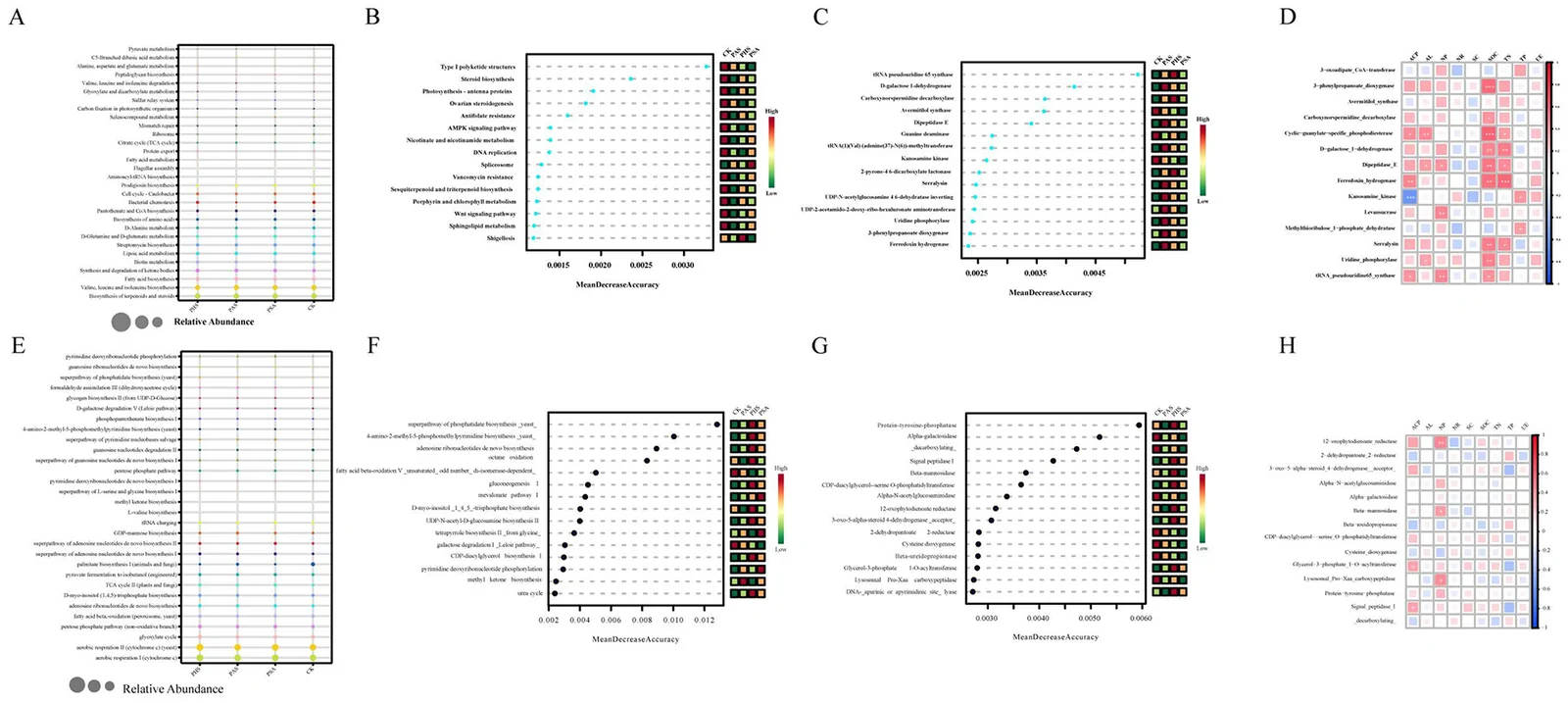
**(A–D)** Functional prediction enzyme and metabolic pathway analysis charts of bacterial communities; **(E–H)** functional prediction enzyme and metabolic pathway analysis charts of fungal communities.

Functional prediction analysis was performed on soil microbial communities under the four planting patterns (Figure 10). Based on abundance bubble plots and random forest analysis, several relatively important KEGG pathways were comprehensively identified across the four treatments. Among the bacterial communities, Metabolism of terpenoids and polyketides (Type I polyketide structures) was identified as an important KEGG pathway. In the fungal communities, the Urea Cycle was identified as the key KEGG pathway. Enzymes showing significant differences in relative abundance among treatments were further screened, and their correlations with soil properties were analyzed. The results showed that, in soil bacterial communities, the relative abundances of Levansucrase and Ferredoxin hydrogenase were correlated with soil-related indicators. In soil fungal communities, the predicted relative abundances of β-mannosidase and lysosomal Pro-Xaa carboxypeptidase were also correlated with soil properties.

## 4 Discussion

### 4.1 Differential effects of intercropping patterns on soil physicochemical properties and enzyme activities

The present study showed that the introduction of different medicinal plants into *P. elliottii* plantations led to marked changes in soil nutrient status and enzyme activities, and that these changes were characterized by clear species specificity and soil-depth dependence. In the 0–20 cm soil layer, the PSA treatment had significantly higher TP contents than the pure P. elliottii stand (CK), a trend that may be related to phosphorus activation of insoluble soil phosphorus in acidic lateritic red soil driven by root exudates and litter input from Alpinia hainanensis within the PSA system (Kuzyakov and Blagodatskaya, 2015; Štursová et al., 2012). In the 20–40 cm soil layer, PHS and PSA had significantly higher TN than CK, and only PHS maintained elevated subsoil SOC, which might be partly attributable to deep fine-root residues of Ficus simplicissima that improved deep soil carbon retention. All treatments showed prominent surface enrichment of SOC and TP, as low phosphorus mobility in subtropical red soils restricts phosphorus cycling to topsoil (Vitousek et al., 2010).

The two-way ANOVA further showed that intercropping pattern significantly affected S-ACP, UE, and NR activities, whereas its overall effects on TN, TP, and SOC were not significant. This indicates that the effects of medicinal plant intercropping were more consistently expressed through changes in enzyme-mediated nutrient transformation than through uniform changes in soil nutrient.

These enzyme responses may further reflect the regulatory effects of intercropping patterns on nutrient transformation processes. Compared with the pure plantation, specific intercropping treatments increased the activities of acid phosphatase, urease, or neutral protease, all of which are closely associated with nitrogen and phosphorus cycling, whereas amylase and sucrase activities decreased under some treatments. This finding suggests that different combinations of medicinal plants exert distinct influences on soil carbon-source utilization and nutrient acquisition. Acid phosphatase is an important indicator of organic phosphorus mineralization, and an increase in its activity usually suggests a greater phosphorus demand by soil microorganisms and plants or an insufficient supply of available phosphorus, which in turn induces the secretion of related enzymes (Nannipieri et al., 2012). Previous studies have also shown that under-forest mixed planting can significantly enhance soil enzyme activities and improve nutrient mineralization efficiency by altering litter quality and rhizosphere carbon inputs (Beule and Bachmann, 2022; Guo et al., 2025).

It is noteworthy that the effects of different intercropping patterns on enzyme activities were not consistent. This suggests that the beneficial effects of intercropping systems are not determined simply by species number, but rather by the combined influence of plant functional traits, root distribution patterns, and litter quality. In other words, medicinal plant combinations with stronger capacities for carbon sequestration, nitrogen mineralization promotion, and phosphorus activation are more likely to create a microenvironment favorable for nutrient cycling in *P. elliottii* plantations. This conclusion is consistent with the view that diversified plant assemblages can promote nutrient complementarity and improve resource-use efficiency (Forrester and Bauhus, 2016).

This set of variations in soil nutrients and enzyme activities shows that intercropping specific medicinal herbs under *P. elliottii* was correlated with improved soil nutrient pools and enzyme activity, which may link to the formation of beneficial soil microecological feedback.

### 4.2 Ecological trade-offs of intercropping patterns and functional differentiation along the soil profile

The two-way ANOVA identified soil depth as the primary factor affecting soil properties, with highly significant effects on TN, SOC, AL, S-ACP, NP, and UE. Consistent with these results, SOC and several soil enzyme activities tended to be higher in the 0–20 cm layer than in the 20–40 cm layer, indicating pronounced vertical differentiation in soil nutrient cycling and enzyme-mediated processes. Surface soil may be more strongly influenced by litter inputs and rhizosphere activity, whereas deeper soil is more closely associated with root-derived carbon inputs and nutrient redistribution. In the PSA treatment, the relatively high TN content in the 20–40 cm layer may be associated, to some extent, with the enrichment of *Sphingomonas*. Previous studies have shown that *Sphingomonas* taxa generally possess strong environmental adaptability and are capable of degrading a wide range of aromatic and complex organic compounds; therefore, their enrichment may be related to organic matter transformation and nitrogen turnover in deeper soil layers.

From the perspective of ecological trade-offs, intercropping systems may raise soil organic matter input and nutrient cycling capacity, which could correlate with elevated microbial activity and improved soil fertility. Complex positive and negative interactions commonly coexist within mixed agroforestry plantations, a classic ecological trade-off widely summarized in previous theoretical research (Jose, 2009; Cannell et al., 1996). In addition, the differences among treatments across soil layers indicate that the effects of intercropping on soil functioning are not spatially uniform, but are likely co-regulated by root distribution, litter decomposition rates, and soil hydrothermal conditions. For example, herbaceous medicinal plants usually possess shallow and dense fibrous root systems that can potentially accelerate nutrient cycling in the surface soil, whereas vine species or deep-rooted plants may affect deeper soil through root penetration and the input of root-derived exudates. Similar profile differentiation has also been widely observed in agroforestry systems, where under-forest intercropping can significantly alter soil aggregates, enzyme activities, and microbial distribution at different depths, thereby potentially generating stronger vertical heterogeneity in belowground ecological processes (Udawatta and Jose, 2011; Beule and Bachmann, 2022). Therefore, from a management perspective, evaluating the effects of medicinal plant intercropping in forest plantations should not be limited to the surface soil alone; rather, nutrient transformation and carbon pool responses across different soil profiles should also be considered in order to more comprehensively assess its ecological benefits.

### 4.3 Intercropping-associated changes in soil microbial community structure and predicted functions

Microbial communities are among the most sensitive ecological components responding to medicinal plant intercropping systems. In the present study, all intercropping patterns significantly altered the structure of both bacterial and fungal communities and generally increased microbial diversity and network complexity relative to the pure *P. elliottii* plantation, which may be associated with changes in soil resource availability and belowground niche conditions. In particular, the PSA treatment showed relatively high bacterial Chao and Sobs indices, and this pattern may be associated with more diverse resource inputs and heterogeneous soil microhabitats that support a greater number of microbial taxa. This finding is consistent with the widely recognized positive relationship between plant diversity and microbial diversity (Bardgett and van der Putten, 2014; Fierer, 2017).

In terms of community composition, the intercropping treatments reduced the relative abundance of oligotrophic taxa such as Acidobacteriota, while enriching certain microbial groups associated with organic matter decomposition and plant growth promotion, including *Sphingomonas*. This trend may reflect a potential transition of the soil system from a “low-resource, slow-turnover” state toward a “high-resource, fast-turnover” state. Acidobacteriota are typically dominant in low-pH, nutrient-poor environments, and a decline in their relative abundance is often regarded as an indicator of improved soil fertility and increased availability of labile carbon sources (Fierer et al., 2007; Kielak et al., 2016). At the same time, taxa such as *Sphingomonas* are known for their strong potential to degrade organic pollutants, their broad environmental adaptability, and their possible roles in potential plant-associated functions and ecological remediation under stressed conditions (Kertesz and Kawasaki, 2010). Therefore, different intercropping patterns not only altered the abundance of microorganisms, but more importantly shifted their functional orientation.

The network analysis further demonstrated that intercropping enhanced microbial synergistic interactions. Compared with the pure plantation, the co-occurrence networks under all intercropping treatments were generally dominated by positive correlations, a pattern that may be associated with microbial communities shaped mainly by mutual coexistence or niche differentiation rather than intense interspecific competition. Networks dominated by positive correlations usually imply more sufficient resource supply, stronger environmental filtering, and greater internal cooperation within the community (Faust and Raes, 2012; Banerjee et al., 2018). Among the treatments, the bacterial network in the PHS pattern exhibited a relatively high clustering coefficient and robustness, suggesting a more complex interaction structure and greater system stability, which may help maintain soil functional redundancy and resistance to disturbance. This increase in network-level stability implies that medicinal plant intercropping can not only increase species richness, but may also enhance soil ecosystem stability by promoting coordination among functional groups.

Collectively, shifts in microbial community composition, diversity and co-occurrence network stability may be related to divergent microecological performances among distinct forest-medicine intercropping regimes.

## 5 Conclusions

This study mainly investigated the effects of intercropping different medicinal plants in *P. elliottii* plantations on soil nutrient contents, enzyme activities, and microbial community structure. The results showed that medicinal plant intercropping exerted clear improvement effects on *P. elliottii* plantations. Intercropping increased the contents of soil TN, TP, and SOC, particularly under *P. elliottii*–*A. hainanensis*–*S. suberectus* (PSA) pattern. Two-way ANOVA further showed that soil depth was the primary factor affecting TN, SOC, AL, S-ACP, NP, and UE, whereas intercropping pattern mainly affected S-ACP, UE, and NR. Significant intercropping pattern × soil depth interactions were detected only for AL and NP, indicating that the responses of these two enzyme activities were depth-dependent. Intercropping also significantly enhanced soil microbial richness and altered community structure. The PSA treatment exhibited the highest ASV richness for both bacteria and fungi, and its bacterial Chao and Sobs indices differed significantly from those of the CK treatment. In addition, the relative abundance of Chloroflexota was higher in all three intercropping treatments than in CK. Microbial co-occurrence network analysis further showed that the stability of microbial communities under all intercropping patterns was higher than that in CK. In the fungal network, the PAS treatment showed the highest stability. Across all treatments, microbial networks were dominated by positive correlations (over 95%) and exhibited relatively high modularity, indicating that intercropping promoted synergistic interactions among microorganisms. Overall, the *P. elliottii*–*A. hainanensis*–*S. suberectus* (PSA) pattern caused substantial changes in soil nutrient status, enzyme activities, and microbial community structure, and therefore may be considered a promising intercropping pattern for application in *P. elliottii* plantations to support their sustainable management.

## Data Availability

The data presented in this study have been deposited in the NCBI Sequence Read Archive (SRA) under accession number PRJNA1464333 and PRJNA1464371.
